# Supplementary material for: Using patient‐specific phantoms to evaluate deformable image registration algorithms for adaptive radiation therapy
Source: J Appl Clin Med Phys. 2013 Nov 4;14(6):177–94. doi: 10.1120/jacmp.v14i6.4363 (PMC4041490; doi:10.1120/jacmp.v14i6.4363)
Supplement: Supplementary file 1 — Supplementary Material [file ACM2-14-177-s001.doc]

Using Patient-Specific Phantoms to Evaluate Deformable Image Registration Algorithms for Adaptive Radiation Therapy

Nick Stanley, Carri Glide-Hurst, Jinkoo Kim, Jeffrey Adams, Shunshan Li,

Ning Wen, Indrin Chetty, Hualiang Zhong

Department of Radiation Oncology

Henry Ford Health System, Detroit, MI

Corresponding Author:

Hualiang Zhong

Department of Radiation Oncology

Henry Ford Health System

2799 W Grand Blvd.

Detroit, MI, 48202

Email: hzhong1@hfhs.org

Phone: 1 (313) 874-3954

Fax: 1 (313) 916-3264

Conflict of Interest: None.

**Abstract:**

**Purpose:** The quality of adaptive treatment planning depends on the accuracy of its underlying deformable image registration (DIR). The purpose of this study is to evaluate the performance of two DIR algorithms, B-Spline based deformable multi-pass (DMP) and deformable demons (Demons), implemented in a commercial software package.

**Methods:** Evaluations were conducted using both computational and physical deformable phantoms. Based on a finite element method (FEM), a total of eleven computational models were developed from a set of CT images acquired from four lung and one prostate cancer patients. FEM generated displacement vector fields (DVF) were used to construct the lung and prostate image phantoms. Based on a fast-Fourier transform technique, image noise power spectrum was incorporated into the prostate image phantoms to create simulated CBCT images. The FEM-DVF served as a gold standard for verification of the two registration algorithms performed on these phantoms. The registration algorithms were also evaluated at the homologous points quantified in the CT images of a physical lung phantom.

**Results:** The results indicated that the mean errors of the DMP algorithm were in the range of 1.0~3.1 mm for the computational phantoms and 1.9 mm for the physical lung phantom. For the computational prostate phantoms, the corresponding mean error was 1.0-1.9 mm in the prostate, 1.9-2.4 mm in the rectum and 1.8-2.1 mm over the entire patient body. Sinusoidal errors induced by B-spline interpolations were observed in all the displacement profiles of the DMP registrations. Regions of large displacements were observed to have more registration errors.

**Conclusions:** Patient-specific FEM models have been developed to evaluate the DIR algorithms implemented in the commercial software package. It has been found that the accuracy of these algorithms is patient dependent, and related to various factors including tissue deformation magnitudes and image intensity gradients across the regions of interest. This may suggest that DIR algorithms need to be verified for each registration instance when implementing adaptive radiation therapy.

**Keywords:** deformable image registration, validation, finite element modeling, deformable phantom

Conflict of Interest: None

**1. Introduction**

Anatomy revealed in a planning CT image may change during the course of radiation treatment due to factors such as patient breathing, set up errors or patient weight loss ^1,2^. Anatomical changes may compromise the accuracy of dose calculation for each organ. As a result, the treatment plan developed may not provide necessary target coverage and organ-at-risk sparing. Adaptive radiotherapy (ART) aims to minimize the dosimetric impact of anatomical changes by re-optimizing the original treatment plan if its quality degrades^3^. A key step in the implementation of ART is to match each point on daily CT images to their correspondent points in the planning image. This process can be accomplished with deformable image registration (DIR) techniques ^4^. DIR is to derive a transformation map by maximizing the intensity similarity between the two images being registered. Depending on the registration techniques used, the transformation map can be represented by different mathematical models such as affine transform^5^, thin-plate spline^6^ or B-spline basis^7^, or adapted directly through optical flow-based equations^8^. Similarity metrics also can be represented in different forms including the sum of squared difference, cross-correlation, or normalized mutual information^9,10^. Like dose calculation algorithms, these DIR algorithms must be thoroughly evaluated before they are used in clinic for adaptive radiation therapy.

Visual evaluation is common practice after performing an image registration. As a preliminary test, this evaluation is convenient and especially useful for software development ^11,12^. Along this direction, many efforts have been made using landmarks or contours to estimate errors in the displacement vector field (DVF)^13,14^ of DIR. For example, Hardcastle *et al.* evaluated two registration algorithms with dice scores calculated on the contours drawn by physicians ^15^, and Brock *et al.* ^16^ evaluated 4DCT registrations reported from 21 institutions by comparing the computer-predicted displacement at each bifurcation point with the displacement computed from the oncologists’ annotations. Castillo *et al*. developed an automatic method to identify and track landmark points in lung patient datasets^17^. These studies provided quantitative evaluation results on the performances of different DIR algorithms at these distinctive landmarks or their nearby regions. However, as reported by Kashani *et al*.^18^ and Liu *et al.*^19^, large registration errors can be observed in regions of uniform image intensity, and the above evaluations are limited by the number of the objects being tracked, so errors estimated by the feature-guided evaluation methods may not be representative of the registration accuracy in voxels at a distance from those landmarks^20^.

Since landmarks or contours are not always available in high dose gradient regions, mathematical properties of the generated deformation maps may serve as an alternative metric to evaluate the quality of DIR in these regions. For example, Schreibmann *et al.* evaluated the quality of image registrations by calculating the curl of their deformation maps ^21^; The authors proposed the concept of unbalanced energy, calculated directly from DVF, to detect DIR errors^22^; Bender and Tome employed consistency metrics to evaluate the accuracy of the composed deformation maps; and Klein *et al.* used permutation and ANOVA tests to compare the relative performance of 14 nonlinear registration algorithms ^23^. These studies help evaluate the overall quality of different registrations and the derived information is valuable to clinic. However, as radiation dose was mapped by the derived DVF, sub-voxel displacement errors may cause the accumulated dose over- or underestimated, especially when the dose was counted on individual particles during the dose mapping process^24,25^. Salguero *et al.* demonstrated that registration errors greater than 1 mm can induce large dose errors in high dose gradient regions ^26^, so the accuracy of the registration algorithm needs to be quantified at each image voxel in these regions. In addition, the performance of a registration algorithm should be evaluated for patient-specific registration scenarios that may involve different image qualities, anatomy patterns (e.g. size of homogeneous organs), and deformation magnitudes.

Computational modeling can help evaluate registration performance under various simulated scenarios ^27,28^. For example, Wang *et al.* ^29^ used a set of B-Spline-based mathematical phantoms to evaluate their demons algorithm, and Liu *et al.* ^19^ evaluated their DIR algorithms using the computational phantom NCAT. While the NCAT phantom has organs assigned with uniform intensity, the deformation of each organ was realistically modeled with B-Spline functions. Furthermore, DIR algorithms could be verified with more realistic images acquired from deformable physical phantoms ^19,30-32^. Recently Nie *et al.* used a set of computer-simulated deformable phantoms and a physical pelvic phantom to evaluate different DIR algorithms, and demonstrated the impact of different deformations on the performance of these algorithms^33^. Their computational phantoms were generated by the commercial software ImSimQA. The global deformation of these phantoms was interpolated from the thin-plate splines which were guided by a set of manually selected control points^34^. Consequently, mass volume may not be preserved during the spline interpolation, and the resultant organ deformation may not be physically realistic.

In this study, we will first develop more realistic computational phantoms from CT images of lung and prostate patients. The images of the prostate phantoms were enhanced by the simulated CBCT noise. The reality of the phantom deformations is achieved through an in-house developed finite element modeling framework where tissue elasticity and volume change in each element were characterized by Young’s modulus and Poisson ratio, and the global deformation was controlled by the conservation of tissue elasticity energy and external work. Taking these phantoms as a ground truth, we will investigate parameter settings for the DIR algorithms implemented in Velocity Advanced Imaging (VelocityAI), a commercial DIR software package, and compare the performance of these DIR algorithms for five cancer patients under different deformation scenarios. In addition, we will also develop a motor-controlled deformable physical phantom to verify these algorithms.

**2. Materials and Methods**

**2.1. Image registration software package**

The VelocityAI software package (v2.6.2, Velocity Medical Solutions, Atlanta, GA) offers a rigid registration and three choices for 3D deformable registration algorithms: “Deformable Demons” (Demons), “Deformable Single-Pass” (DSP), and “Deformable Multi-Pass” (DMP). DMP uses DSP with preprogrammed choices for grid settings for each pass. Specifics of the grid settings are unknown to the authors as VelocityAI is commercial, proprietary software. However, it is known that DMP uses mutual information as its similarity metric and B-splines for interpolation. Operators also have the option of limiting the registration space to a region of interest (ROI). Registration can be performed intra and inter-modality for CT, CBCT, and MRI, and its derived DVF can be exported as a binary file with 3-dimensional values for each voxel.

The purpose of this study is to evaluate the DMP and Demons algorithms implemented in VelocityAI. Different from DMP, DSP allows users to choose different grid settings ranging from “coarse” to “fine”. However, due to the lack of a ground truth, these settings are hardly to be used in clinic. In this study, we will first use the developed computational phantoms to evaluate different grid settings for DSP and compare their registration results with those derived from the DMP algorithm, and then we will focus on the comparison between DMP and Demons for different scenarios of organ deformation.

**2.2. Evaluation of image registration algorithms with lung CT images**

**2.2.1 Development of computational lung phantoms**

### 4DCT datasets used in this study were selected from four lung cancer patients under a retrospective protocol approved by the **Institutional Review Board** of our institution (IRB#: 6203). For each case studied, the 3D image of the 4DCT at the end inhalation (EI) phase was considered the primary image. Computational phantoms were developed based on the primary images using a finite element modeling (FEM) system. The mathematical implementation of this system was described in our previous study^28^. With this system, tetrahedral meshes were generated and scaled to match each of the image domains. Diaphragm, spinal cord, and ribs on each side were manually segmented from the CT images. Tetrahedral nodes located in diaphragm regions were selected as driving nodes, and those in spinal cords and side ribs were fixed as boundary constraints. Young’s moduli were set to 1 MPa for ribs, 1 kPa for lung, and 10 kPa for other soft tissue. The Poisson ratio was 0.38 for lung and 0.49 for other elements^35^. With different forces assigned to the driving nodes, the displacement vectors of other anatomical structures were computed using the FEM modeling system.

For lung patient 1 (denoted Lung1), the selected nodes were assigned with the forces of 0.8, 1.6, 2.4 and 3.2 kPa, and these forces got the diaphragm moved superiorly by about 1, 2, 3 and 4 cm. The force-induced organ deformation can be visualized through the overlay of the patient’s original image set and its deformed image set shown Figure 1. For lung patient 2 (denoted Lung2), the forces of 0.8, 1.2 and 2.0 kPa caused diaphragm deformation by 1.8, 2.7 and 4.2 cm, respectively. These phantoms will be used to evaluate the impact of motion magnitudes on registration accuracy.

To evaluate the impact of patient-specific anatomy on DMP registrations, CT images of two additional lung patients (Lung3 and Lung4) were included to develop more computational phantoms. 1.8 and 2.1 kPa forces were assigned to the tetrahedral nodes located in their diaphragm regions, and their lateral ribs and spinal cords were kept stationary as boundary conditions. As a result, their simulated diaphragm deformation is limited to 3 cm. Consequently, there are total four phantoms, including Lung1 and Lung2, having their diaphragms deformed about 3cm.

The displacement vectors of the tetrahedral nodes generated by the FEM modeling system were interpolated to generate a DVF for each primary image. Secondary images were constructed from the primary image sets using the FEM-generated DVFs. Slice spacing and image size were kept consistent with the primary image sets with voxel sizes of ~1 mm in the x and y directions, and 3 mm in the z direction. The FEM-generated DVFs were considered the gold standards to evaluate registrations performed from the primary images to the FEM-simulated images.

**2.2.2 Computational phantom-based evaluations of DSP with different grid resolutions**

DSP and DMP share the same registration algorithm with their difference mainly in that DSP allows the user to choose the B-spline grid resolution from 1 to 9, with 1 being the coarsest and 9 being the finest. The exact grid spacing of each level is unknown due to the proprietary nature of the software. One can compose a set of DSP registrations to form a multi-resolution registration chain. For example, images would be registered first using the coarsest setting, and then that result would be fed to the next stage of finer grid spacing.

In order to evaluate the DSP registration algorithm, chains of registrations with different grid resolutions were performed on the two lung patients (Lung1 and Lung2) with four diaphragm deformations (1.8 cm, 2.0 cm, 2.7, and 3.0 cm). For each dataset, six representative compositions of typical grid resolutions were investigated, including three single grid resolutions of 1, 5, and 9 as well as three resolution compositions of $1\to5$, $1\to9$, and $1\to5\to9$. The resulting DVFs were compared against the corresponding FEM-generated gold standard DVFs to estimate the registration errors at each image voxel. The DMP algorithm was also applied and evaluated using the same dataset for comparison.

**2.2.3. Computational phantom-based evaluation of DMP and Demons**

To evaluate the DMP algorithm which was suggested as the default by the manufacturer, the performances of DMP and Demons registrations were compared based on the developed FEM models. Both DMP and Demons in VelocityAI had no customizable grid settings and was run with the default parameters. Their registrations were performed by deforming the primary image sets to match the secondary image sets. The registration error *e* at a point *x* was quantified by $e(x)=\left|DVF_{FEM}(x)-DVF_{DIR}(x)\right|$, where the first and second terms are the DVFs generated by the FEM modeling and DIR algorithms, respectively.

Both DMP and Demons registrations were performed on seven deformable models, developed from Lung1 and Lung2 to identify the potential trend of registration errors that are associated with different displacement magnitudes; then the two algorithms were evaluated with models developed from four different patients to detect any patient-specific errors. Registration errors are generally large in the superior and inferior ends of image boarders because there is often no corresponding anatomy between two input images. However, the effect is clinically insignificant since the treatment lesions and relevant organs of interest are typically located near the center of the images. Thus, such boarder regions (2 cm on each end) were excluded from analysis in this study. Boolean masks of the patient body and lung region were created to confine the error analysis to these relevant voxels.

**2.2.4. Physical phantom-based evaluation of DMP and Demons**

A motor-controlled lung phantom was developed to simulate respiration-induced deformation (Figure 6a). Phantom characterization is beyond the scope of this work; but briefly, the insert was made of heterogeneous sponge with average density equivalent to lung density in CT images. The inferior end of the phantom was deformed 2.5 cm to simulate a diaphragm movement. A 4DCT dataset including the phases of the end inhalation (EI) and end exhalation (EE) was acquired for this phantom. Registrations were performed from EI to EE using both DMP and Demons algorithms. Registration errors were quantified with the ROIs set to the lung region as well as to the entire image volume. Further, the influence of different window/level settings was investigated. The selected window settings included 250, 500, 1000 and full range with the levels set to the half of the corresponding window values.

The registrations of the phantom images were evaluated with 37 landmarks. These landmarks were sponge features automatically identified in the phantom’s EE image. Specifically, the image was scanned voxel by voxel until one was found with intensity greater than a given threshold. Once this voxel was found, a sub-process utilized a spanning tree algorithm ^36^ to find all the directly neighboring voxels with a new threshold that had lower intensity than the first. The first threshold ensured that only objects whose intensity rose above it would be chosen. The second threshold was set lower to include more periphery voxels and form a complete feature. Once the feature was delineated, its center of mass was determined to define the feature’s spatial location. All voxels within an already defined object were then ignored in the remaining scans.

A deformable image registration was performed from the EI image to the EE image, and its resultant DVF was then used to construct a warped image from the EI image. The process used to identify the features on the EE image was repeated on this warped image. At this point, two sets of features were determined: one on the EE, and another on the warped EI images. The correspondence of these features was established based on their size and center-of-mass coordinates, and was then visually reviewed with their overlaid images. The distance between the corresponding center-of-mass coordinates in the EE image and in the warped EI image was determined, indicating the displacement error of the registration at the center.

**2.3. Evaluation of image registration algorithms with prostate CT images**

**2.3.1. Development of computational prostate phantoms**

The primary CT image sets were acquired from prostate cancer patients using a retrospective protocol approved by the IRB of our institution. One image set with an empty bladder was selected to create a deformable prostate phantom, where bladder expansion was simulated through equally distributed forces applied outward on a set of tetrahedral nodes located in the inside of the bladder. Two different forces were added to these boundary nodes to create two deformed image sets, termed$P_{1}^{CT}$ and $P_{2}^{CT}$, resulting in a 6mm and an 8mm isotropic expansion of the bladder radius in the lateral direction. The resultant displacements of all the tetrahedral nodes were interpolated to get displacements in the entire image domain. The interpolated displacement fields were used to deform the primary image to generate deformable prostate phantoms with known deformation fields.

**2.3.2. Prostate phantoms for evaluation of image registrations between CT and CBCT**

To evaluate DMP performance between CT and CBCT image registrations, simulated CBCT images were created by incorporating simulated CBCT noise into the FEM-generated benchmark models. Specifically, with the two FEM-simulated deformation fields, the primary prostate CT image was warped to create two deformed images. To create simulated CBCT images$P_{i}^{Noise}$, i=1,2, Poisson noise was incorporated into the deformed images in a manner similar to Murphy et al. ^37^. Specifically, the deformed CT image was transformed to its spectrum space using a Fast Fourier Transform (FFT) in MatLab (The MathWorks, Inc.). Based on Jaffray and Siewerdsen’s study of CBCT performance characteristics^38^, the noise power spectrum (NPS) was approximated by the function $p(k)=e^{-k\lambda}(1-e^{-k\lambda})$ with λ=0.15 mm, where k is the wave number in the Fourier domain ^37^. The FFT image was then multiplied by the NPS and converted back to the image domain using an inverse FFT. The resultant image $P_{i}^{Noise}$ contains the simulated CBCT noise. If a registration performed from the primary prostate CT image to $P_{i}^{CT}$ or $P_{i}^{Noise}$ is accurate, its DVF should be equal to the FEM-generated deformation field.

**2.3.3. Evaluation of registrations between prostate CT and CBCT images**

The evaluation of prostate image registrations was performed in the same way as for the lung cases (i.e. a voxel-by-voxel comparison to the FEM simulated DVF for constructed image phantoms). This was done for registrations from the primary CT to $P_{i}^{CT}$ and$P_{i}^{Noise}$, i=1,2, respectively, where $P_{i}^{Noise}$ was used to assess the impact of CBCT noise on image registrations. For this reason, average errors and displacement profiles of these $P_{i}^{Noise}$registrations were compared to the$P_{i}^{CT}$registrations. The calculated errors were averaged in the entire patient volume as well as in the prostate, bladder, and rectum regions.

**3. Results**

**3.1. Evaluation of image registrations using lung CT phantoms**

**3.1.1. Computational lung phantoms**

Figure 1 demonstrates an example for the case of Lung1_2cm_ where the center of the diaphragm surface was moved up by 2 cm and the other internal structures were deformed by the FEM model. The overlay of the original image (red) and the FEM deformed (green) image was shown in Figure 1(a-c). Similarly, computational phantoms were developed for the four lung cancer patients with a set of different deformation magnitudes.


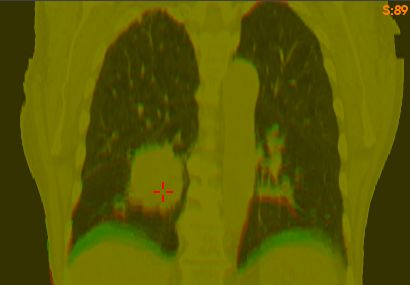

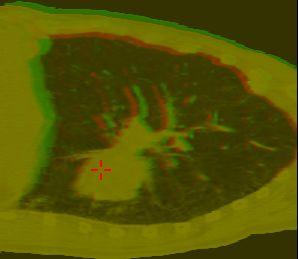

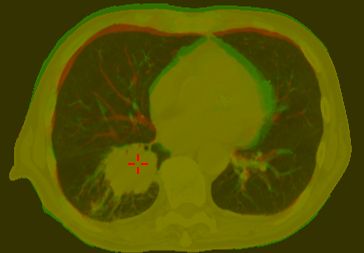


(a) (b) (c)

Figure 1. The original CT image (red) and its deformed image (green) of Lung1_2cm_. (a), (b) and (c) are the coronal, sagittal and transverse cuts of their overlaid images, respectively.

**3.1.2. Evaluation of DSP with different grid settings**

DSP registrations with different B-Spline grid settings were performed on the computational phantoms Lung1_2cm_, Lung1_3cm_, Lung2_1.8cm_, and Lung2_2.7cm_. All the grid settings defined in Section 2.2.2 were tested, and the resultant displacement errors were averaged in the patient volume with the results shown in Figure 2.


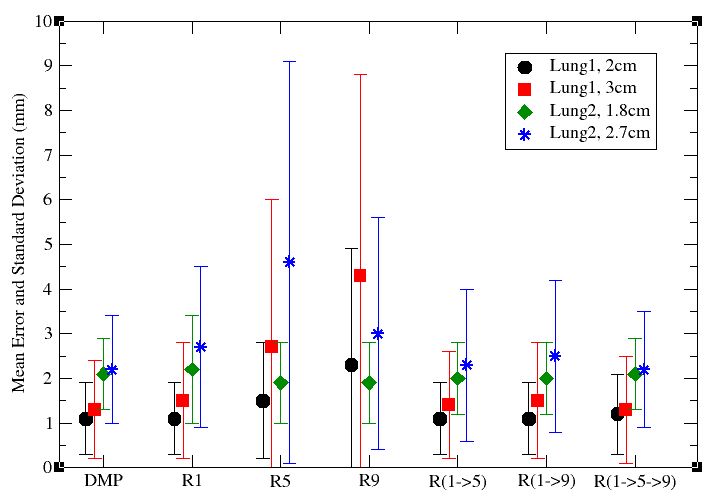


Figure 2. Average displacement errors (± standard deviation) for different B-spline grid settings. R_x_ denotes a DSP registration with the grid resolution x, and R(x🡪y) represents the composition of the DSP registrations with the resolution chain x🡪y.

As shown in Figure 2, DMP outperformed DSP with these selected settings for Lung1_2cm_, while a slight improvement of DSP over the DMP algorithm was observed in Lung2_1.8cm_ where the average displacement error was improved by 0.2mm. For the cases of large diaphragm motions, Lung1_3cm_ and Lung2_2.7cm_, the DMP performed better for all six settings, and in some cases the differences were larger than 3.0mm. In general, the performance of the DMP was comparable or superior to that of the DSP with the selected B-spline grid settings. It is noticeable that, for DSP, the R_1_ resolution registrations have less error than R_9_ registrations, which is particularly true for large deformation cases Lung1_3cm_ and Lung2_2.7cm_. It is primarily due to the nature of the *local* optimization algorithms, where the optimization is trapped in one of the local minima. Therefore, it is suggestive to use low resolution, especially in the presence of large deformation, followed by gradually finer resolutions for detailed anatomy alignments. This may suggest that grid resolutions should be adapted for different patient images as well as different deformation scenarios.

**3.1.3. Evaluation of DMP and Demons registrations**

DMP registrations were performed on the computational phantoms developed from Lung1 and Lung2 at different deformation magnitudes. Figure 3a shows the profiles for the superior-inferior (SI) components of the displacement vectors derived from the registrations of Lung1. These profiles correspond to the SI-line in Figure 3b that passes through the tumor volume of Lung1. The position z=0 mm indicates the most superior slice of the image. It is evident that the DMP displacements are dipping above and below the gold standard sinusoidally due to the B-spline interpolation of the DMP algorithm.


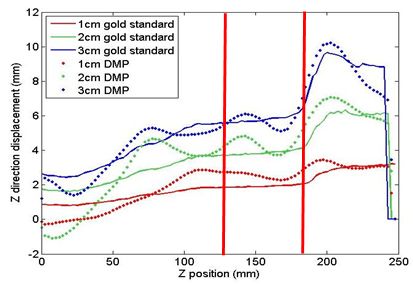

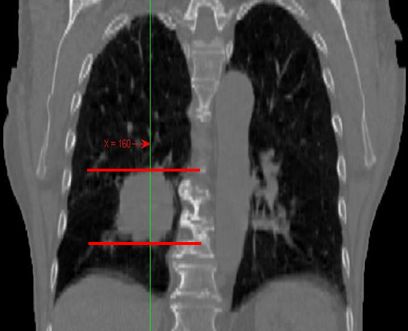


(a) (b)

Figure 3. Superior-inferior (SI) displacement profiles derived from DMP registrations for Lung1. (a) The profiles contain displacements from the gold standard DVFs and DMP DVFs at diaphragm deformation of 1, 2 and 3cm; (b) the SI line illustrated in Lung1’s primary CT image is corresponding to the displacement profiles in (a). The tumor region is marked with two red lines on both the image and profile figures.

The registration errors in the lung as well as in the patient body for DMP and Demons algorithms were summarized in Table 1. It was observed that the mean displacement error was larger for larger diaphragm motion. In all seven cases, the Demons registrations are slightly better than DMP inside the lung, but are much worse outside the lung region for Lung2. The patient-to-patient differences are illustrated further in the next section.

Table 1. Average displacement errors (mm) and standard deviation for DMP and Demons registrations evaluated with computational phantoms of different deformation magnitudes.

| Image set  (motion magnitude) | Patient volume | | Lung | |
| --- | --- | --- | --- | --- |
|  | DMP | Demons | DMP | Demons |
| Lung1 (10 mm) | 1.0 ± 0.7 | 0.8 ± 0.5 | 0.9 ± 0.5 | 0.7 ± 0.4 |
| Lung1 (20 mm) | 1.1 ± 0.8 | 0.9 ± 0.7 | 0.9 ± 0.6 | 0.7 ± 0.4 |
| Lung1 (30 mm) | 1.3 ± 1.1 | 1.1 ± 1.0 | 1.0 ± 0.7 | 0.8 ± 0.6 |
| Lung1 (40 mm) | 1.6 ± 1.5 | 1.4 ± 1.6 | 1.2 ± 1.1 | 1.1 ± 1.2 |
| Lung2 (18 mm) | 2.1 ± 0.8 | 5.2 ± 5.7 | 2.1 ± 0.8 | 2.1 ± 0.9 |
| Lung2 (27 mm) | 2.2 ± 1.2 | 5.2 ± 5.4 | 2.4 ± 1.1 | 2.3 ± 1.2 |
| Lung2 (42 mm) | 3.1± 1.7 | 5.9± 5.6 | 3.3± 2.1 | 3.0± 1.4 |

**3.1.4. Evaluation of DMP and Demons registrations with different patients and regions of interest**

Four patients were simulated with their diaphragm deformed about 3 cm in magnitude to create computational phantoms, and both the DMP and Demons algorithms were applied to these phantoms. The mean errors for the DMP registrations are between 1.3 and 2.6 mm, but the performance of the Demons registrations varies largely among these patients (Table 2).

Table 2. Average displacement errors (mm) and standard deviations for DMP and Demons registrations evaluated with different patient images

| Image set  (motion magnitude) | Patient volume | | Lung | |
| --- | --- | --- | --- | --- |
|  | DMP | Demons | DMP | Demons |
| Lung1 (30 mm) | 1.3 ± 1.1 | 1.1 ± 1.0 | 1.0 ± 0.7 | 0.8 ± 0.6 |
| Lung2 (27 mm) | 2.2 ± 1.2 | 5.2 ± 5.4 | 2.4 ± 1.1 | 2.3 ± 1.2 |
| Lung3 (30 mm) | 2.6 ± 1.7 | 2.1 ± 1.3 | 2.4 ± 1.3 | 1.9 ± 0.7 |
| Lung4 (30 mm) | 2.4 ± 2.3 | 30.0 ± 18.1 | 1.7 ± 1.0 | 9.3 ± 6.3 |

Compared to DMP, Demons has larger errors on the chest wall and breast regions. Inside the lung, Demons has a slightly better performance than DMP in all the cases listed in Tables 1 and 2 except Lung4 (Figure 4), where a lot of homogeneous tissues present a major challenge to the Demons registrations (Figure 4c).


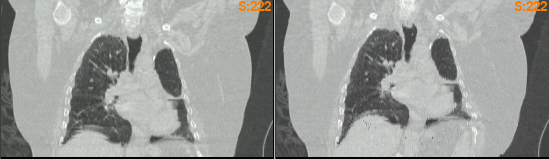


(a) (b)
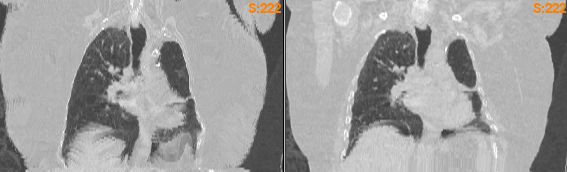


(c) (d)

Figure 4. Computational lung phantom Lung4 with large areas of homogeneous tissue: (a) the original image set, (b) the FEM simulated image set, (c) image warped by Demons DVF, and (d) image warped by DMP DVF.

Even for one patient, different regions could be registered with different qualities. As shown in Figures 5a and 5b, errors from DMP and Demons registrations are overlaid on top of the corresponding image for Lung2_2.7cm_, where large errors from the Demons registration can be observed on chest wall. Similarly, Figures 5c and 5d show the errors of the two registration algorithms overlaid with the coronal cuts of the primary CT image for Lung1_3cm_. Both DMP and Demons have large errors in the regions near the lateral chest wall and the diaphragm as pointed by the arrows in Fig 5c and 5d. These images show the effect of large volumes of homogenous tissue on the respective algorithms.


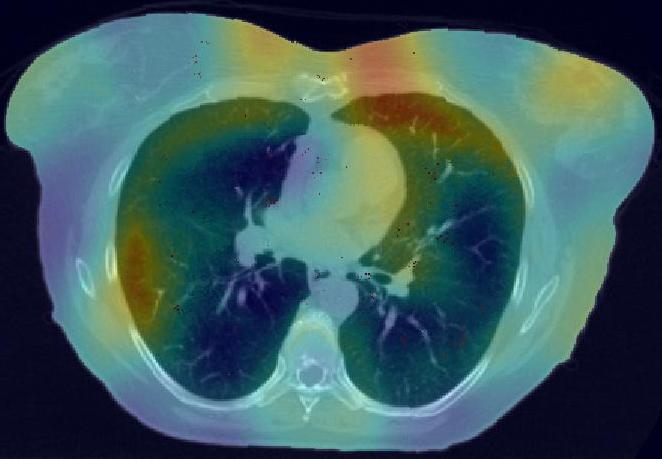

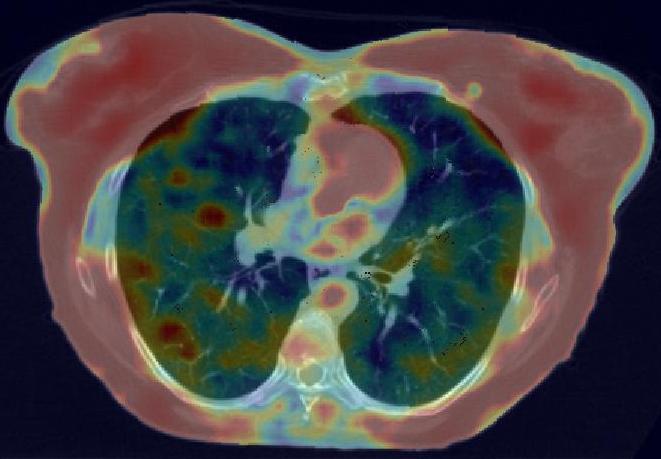


(a) (b)


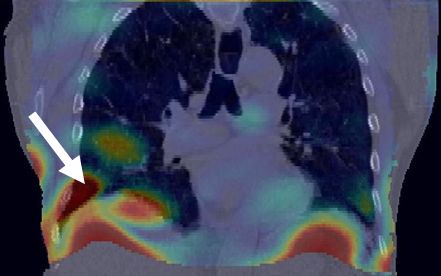

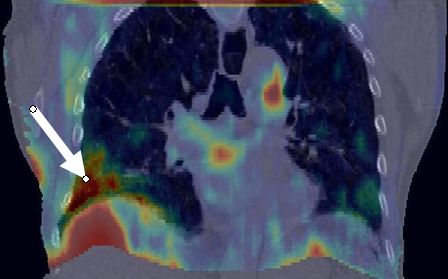

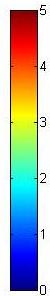


(c) (d) (e)

Figure 5. Color overlay of displacement errors with CT image sets. The figure contains an axial cut of image set Lung2_2.7cm_ overlaid with the errors of (a) DMP registration and (b) Demons registration. Also shown is a coronal slice of Lung1_3cm_ overlaid with (c) DMP and (d) Demons registration errors. Error values seen in the color bar (e) are given in mm.

Overall, the average displacement errors of the DMP and Demons registrations in the lung region ranged from 1 mm to 3.3 mm. In all cases except one, the DMP algorithm produced slightly worse registrations than the Demons algorithm in the lung, but it still provided DVFs with comparable accuracy. There were notable differences between the DMP and Demons registrations outside of the lung region. The Lung2 case demonstrated significantly reduced displacement errors for both the breast tissue and chest wall region when the DMP algorithm was used. Here, displacement errors exceeded 4 cm in the breast tissue for the Demons algorithm but were only 1-2 mm for DMP in the same area.

**3.2. Evaluation of DMP and Demons algorithms with a physical phantom**

DMP and Demons registrations were performed from EI to EE for the physical phantom. The centers of the 37 previously defined landmark objects were automatically tracked. The spatial difference between the centers in the EE image and those in the warped EI image was counted as the registration error. When the registrations were performed over the entire image volume, their average displacement errors were 1.9 and 1.6 mm for the DMP and Demons algorithms, respectively. However, when restricted to the lung regions, the Demons algorithm failed to produce a visually acceptable registration, resulting in more than 10 mm mean error, while the DMP had its average displacement error reduced to 1.8 mm.

Note that the default parameters in DMP were set for patient images which were largely different from the physical phantom. To address this issue, the image intensity window was limited during the DMP registration, based on the manufacturer’s suggestion. Table 3 summarizes average displacement errors for registrations at different window intensity settings. The average of the displacement errors for all the registrations that used customized contrast settings was 0.97±0.51 for the lung region, and 1.25±0.58 for the image domain. The best registrations were in the ROI-constrained registrations with lower window settings,$R_{250}^{ROI}$ and$R_{500}^{ROI}$.

Table 3. Average displacement errors and standard deviations of DMP registrations.

| Mean Error (mm) | DMP registration | | | | |
| --- | --- | --- | --- | --- | --- |
| Intensity Window | 250 | 500 | 1000 | 1500 | Unlimited |
| Image domain | 1.3 ± 0.6 | 1.3 ± 0.5 | 1.1 ± 0.5 | 1.3 ± 0.7 | 1.9 ± 0.8 |
| Lung region | 0.8 ± 0.4 | 0.8 ± 0.4 | 1.2 ± 0.7 | 1.1 ± 0.5 | 1.8 ± 0.6 |

Figure 6 shows the EE image overlaid with the warped EI image using the DMP registrations with the intensity windows of 250 and 500, respectively. The mismatched part of the tumor for the registration$R_{500}^{ROI}$ can be observed in the overlaid images. The results show that the intensity window could impact the DMP registrations, and is worth investigating further in future studies.


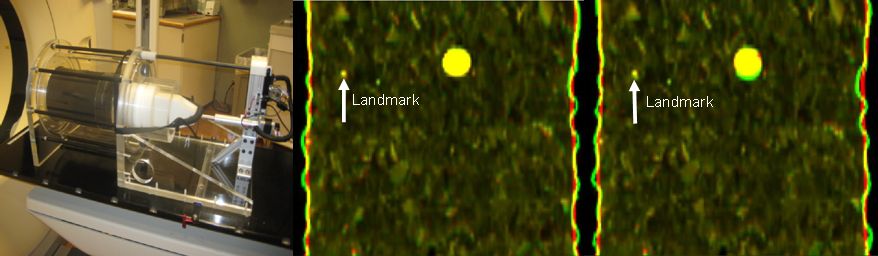


(a) (b) (c)

Figure 6. Physical phantom image sets. (a) A picture of the physical phantom; the overlay of EE and warped EI images registered with (b) 250 window setting and (c) 500 window setting. The green image is the EE image and the red one is the warped EI image. The large circular object represents the tumor.

**3.3. Evaluation of DMP and Demons registrations with prostate CT images**

**3.3.1. Computational prostate phantoms**

Figures 7a and 7b show the original and computationally deformed prostate images. The expansion of the bladder and the deformation of its nearby structures can be observed in the elliptical region marked in the axial and coronal images, respectively. With the method described in section 2.3.2, simulated CBCT noise was incorporated into the deformed image (Figure 7b) to derive a simulated CBCT image (Figure 7c).


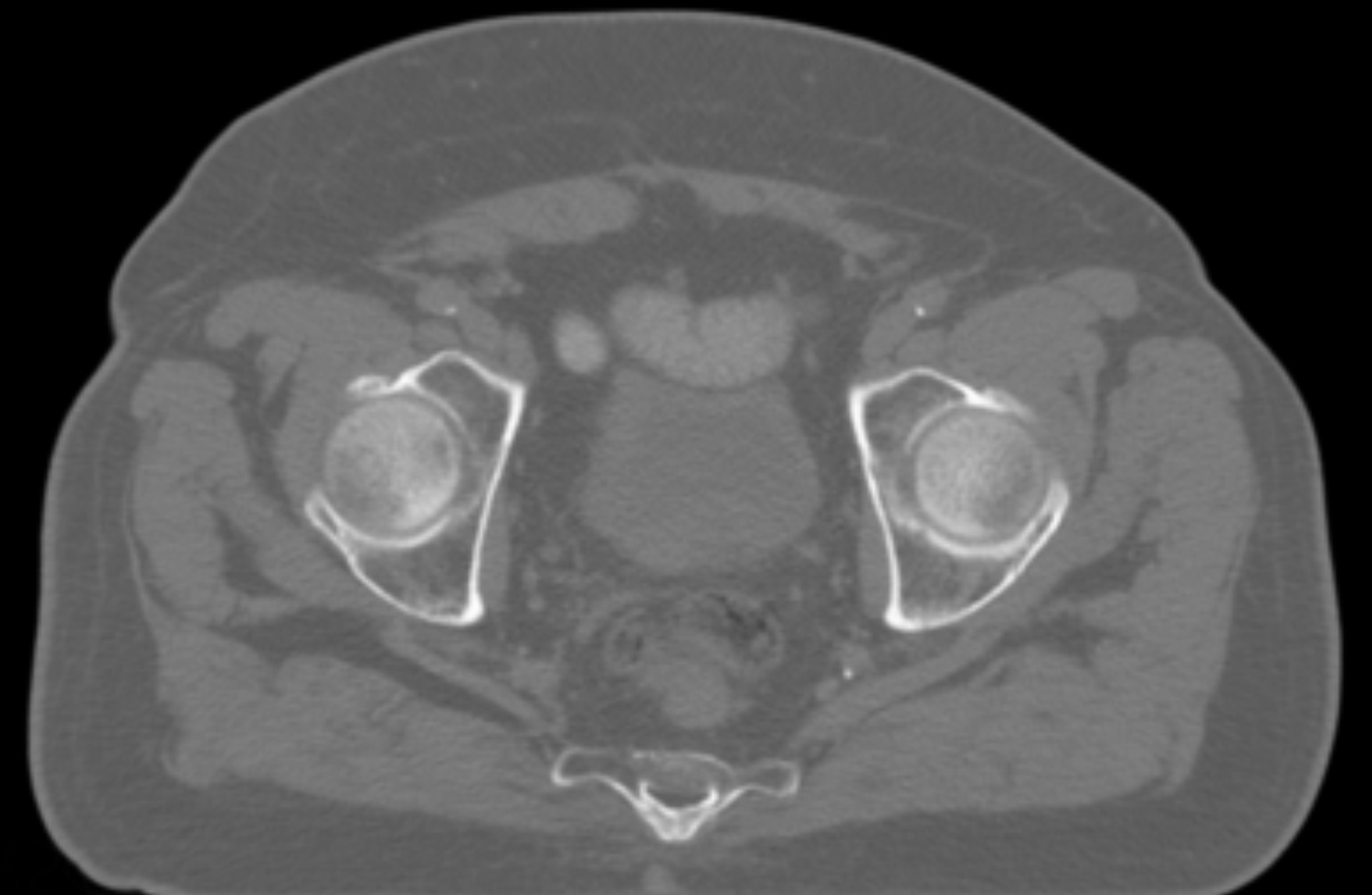

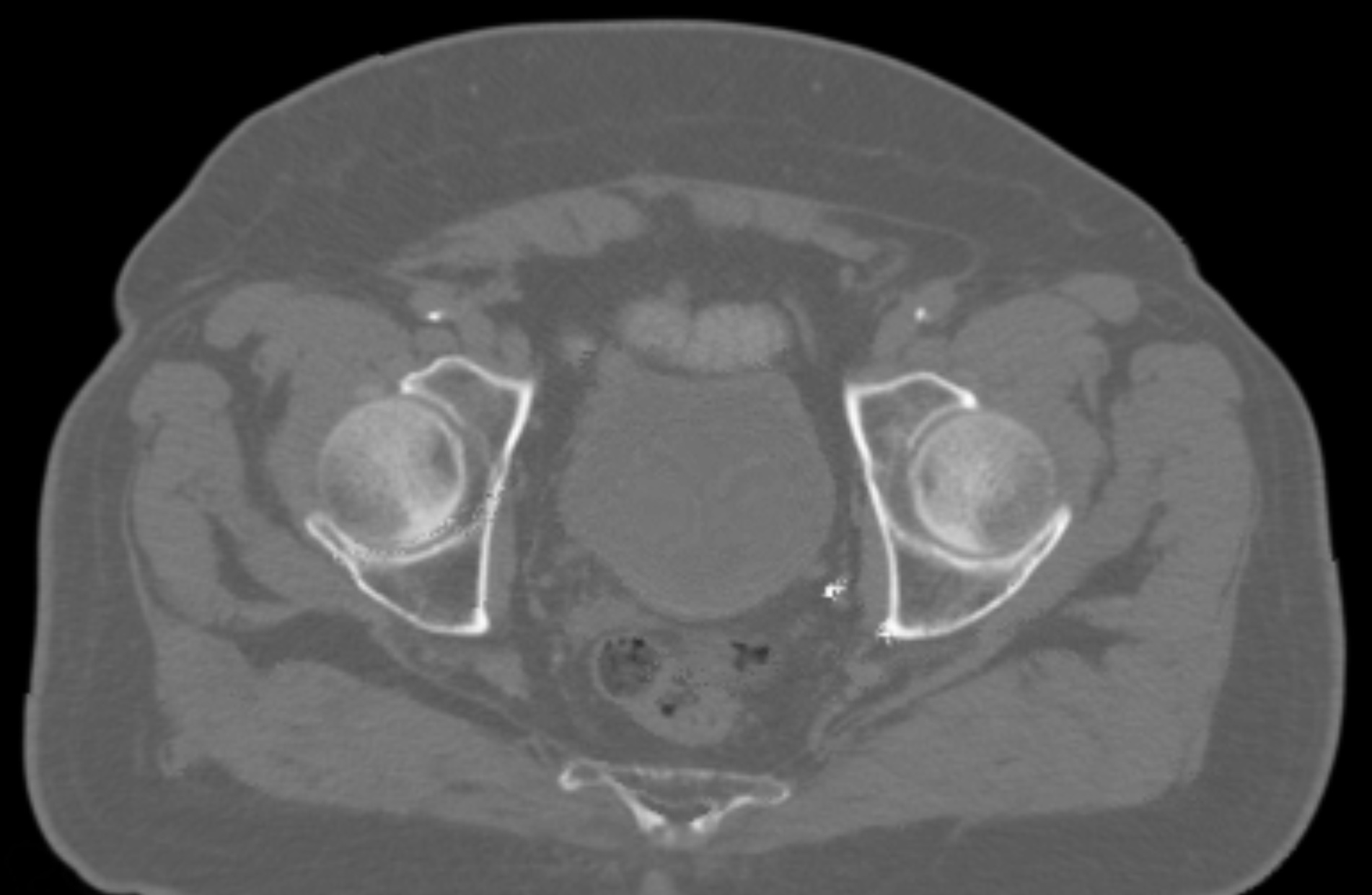

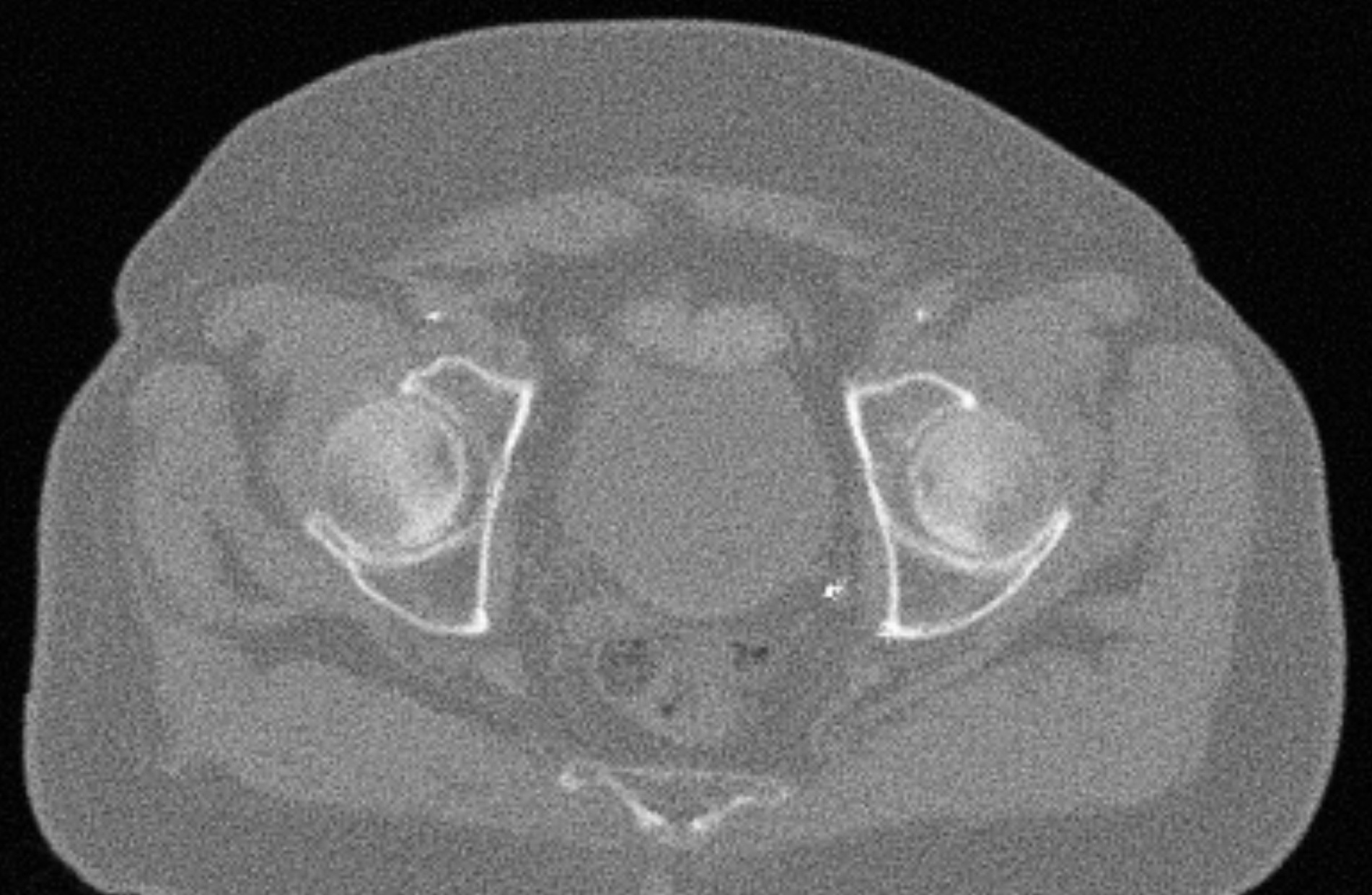


(a) (b) (c)


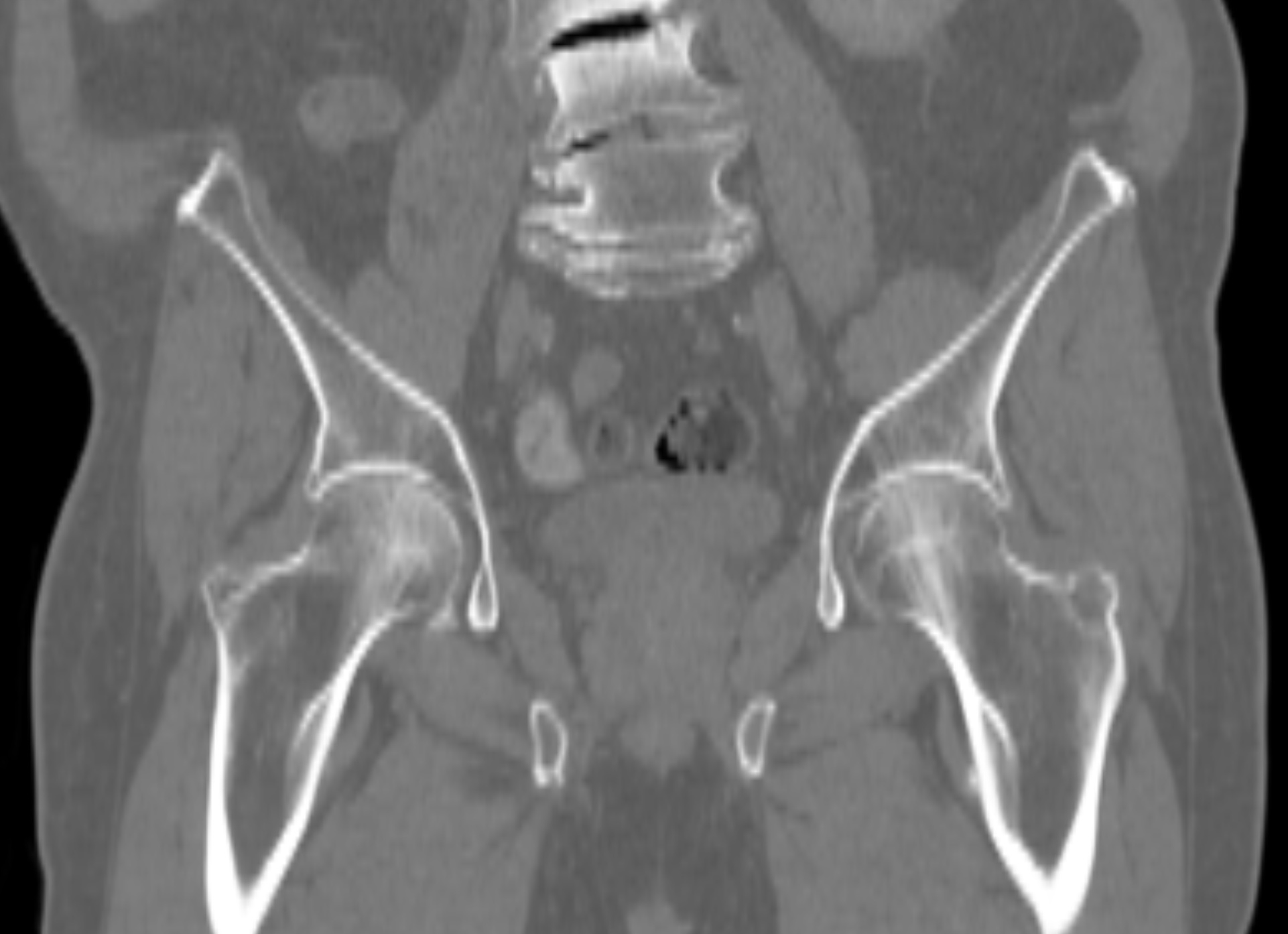

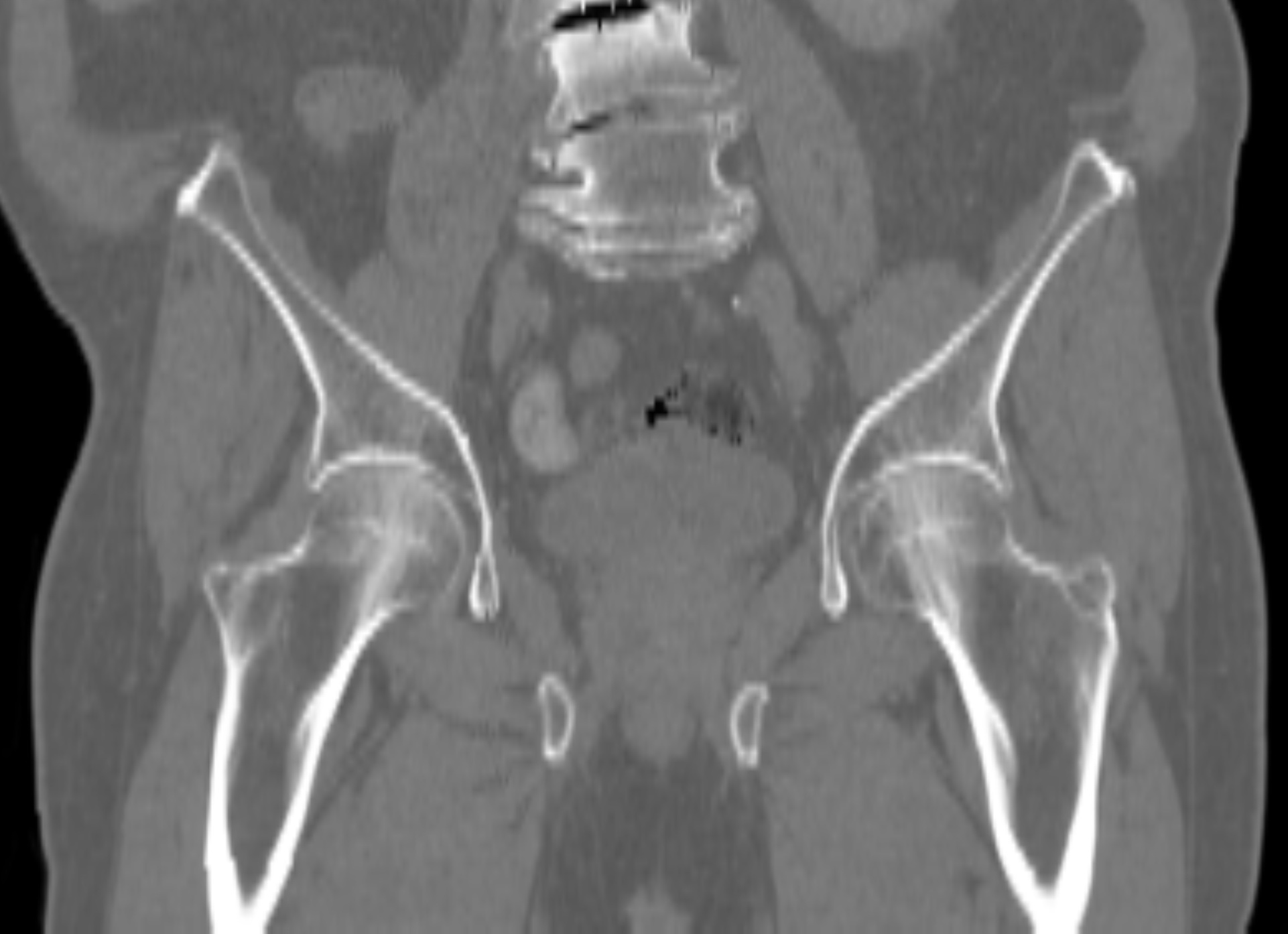

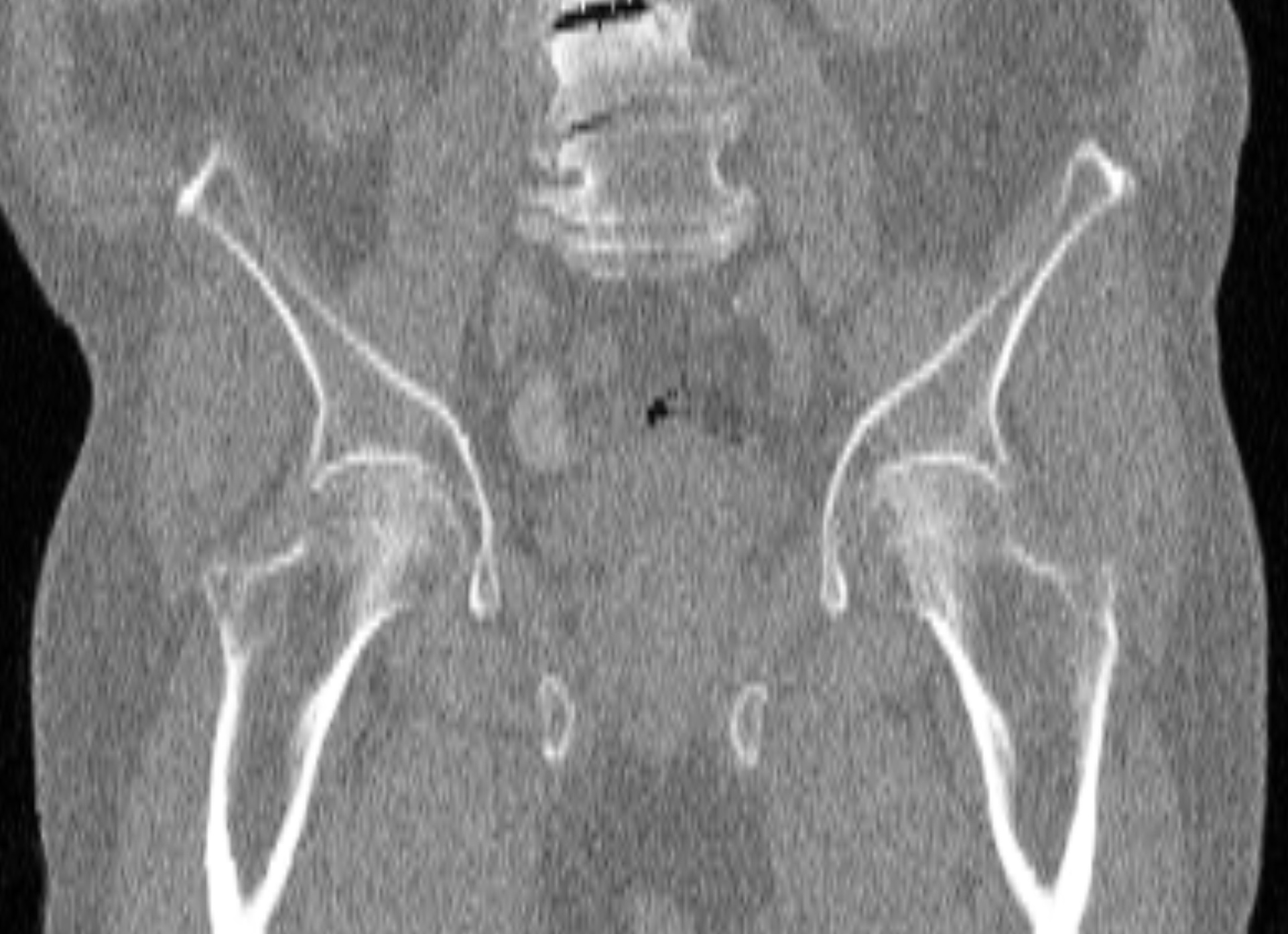


(d) (e) (f)

Figure 7. Computational prostate phantom and simulated CBCT image sets. The axial cuts of (a) the original image set, (b) the deformed image set and (c) the simulated CBCT image. The subfigures (d-f) are the coronal views of (a-c), respectively.

**3.3.2. Evaluation of DMP and Demons registrations using computational prostate phantoms**

Table 4 lists the errors for the DMP and Demons registrations. For DMP, the average registration errors were 1.9 and 2.1 mm across the whole patient volume and 1.2-2 mm for prostate and rectum. The errors in bladder, 4.6 mm on average, were relatively large primarily due to the large initial deformation with low image gradients in the bladder. For the Demons registrations, without noise added, the displacement errors across the whole patient body were large, 10.3 mm and 7.8 mm for $P_{1}^{CT}$ and$P_{2}^{CT}$, respectively (the first and second rows in Table 4). The large errors were mostly observed in the outer tissue regions near the skin surface, while regions of clinical interest, including the prostate and rectum, yielded registration errors between 2.2-3.2mm.

Table 4. Average displacement errors (mm) and standard deviation for DMP and Demons registrations for pelvic images .

|  | Whole Patient | | Prostate | | Rectum | | Bladder | |
| --- | --- | --- | --- | --- | --- | --- | --- | --- |
| Image Set | DMP | Demons | DMP | Demons | DMP | Demons | DMP | Demons |
| $P_{1}^{CT}$ | 2.1 ± 0.7 | 10.3±11.5 | 1.9 ± 0.5 | 3.2 ± 1.2 | 1.9 ± 0.5 | 2.2 ± 0.9 | 2.8 ± 1.5 | 3.7 ± 1.8 |
| $P_{2}^{CT}$ | 1.9 ± 1.0 | 7.8 ± 8.3 | 1.2 ± 0.6 | 2.4 ± 1.3 | 2.0 ± 0.8 | 2.4 ± 1.0 | 4.6 ± 3.1 | 5.0 ± 3.4 |
| $P_{1}^{Noise}$ | 2.1 ± 0.8 | 18.5±18.1 | 1.7 ± 0.5 | 12.3±10.5 | 1.8 ± 0.6 | 12.8±11.2 | 3.1 ± 1.7 | 16.3±13.9 |
| $P_{2}^{Noise}$ | 1.8 ± 0.9 | 19.4±20.7 | 1.0 ± 0.6 | 13.2±11.7 | 2.4 ± 1.0 | 13.3±12.0 | 5.0 ± 3.4 | 18.3±14.5 |

For the simulated CBCT images, the Demons algorithm was not able to produce visually satisfactory results and its registration errors were greatly increased. The impact of the simulated noise on DMP was small with slight variations observed from region to region. Table 4 lists the average displacement errors for all the DMP and Demons registrations.

Figure 8a shows the sagittal view of$P_{1}^{CT}$. Figure 8b and 8c are its corresponding images generated by the DMP and Demons registrations. Their displacement error maps overlaid on $P_{1}^{CT}$are shown in Figure 8e and 8f. The sagittal views illustrate the errors of these registration algorithms in the homogenous fatty tissue as well as higher contrast medial tissues.


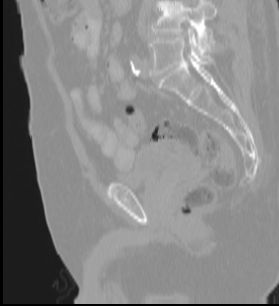

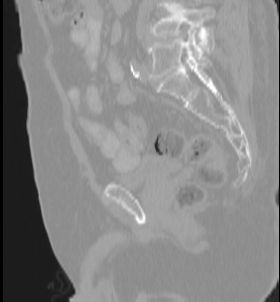

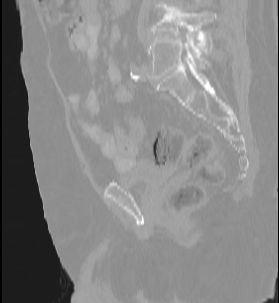


(a) (b) (c)


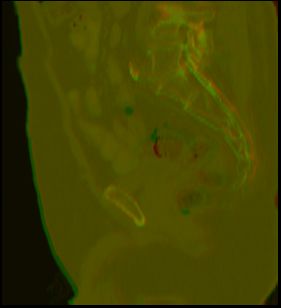

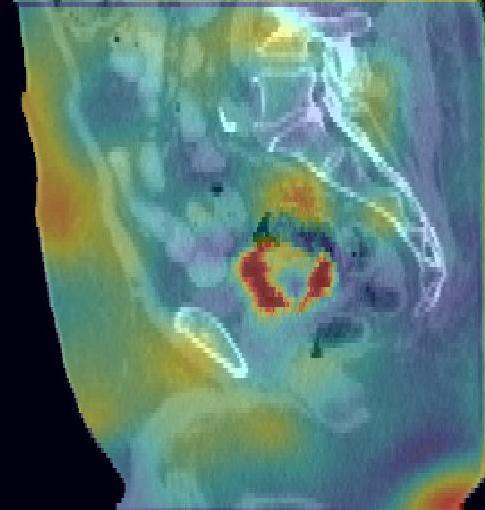

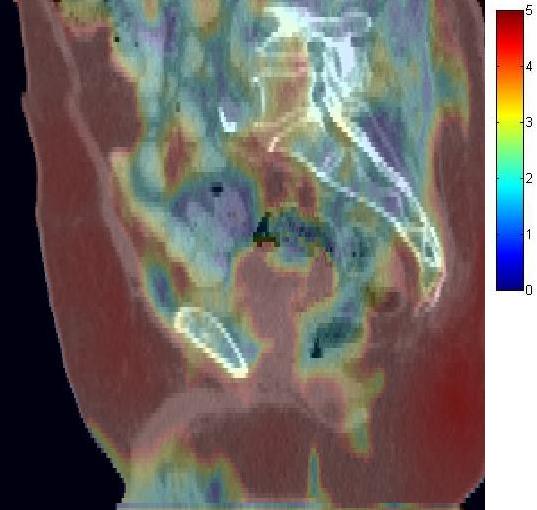


(d) (e) (f)

Figure 8. (a) the FEM constructed image$P_{1}^{CT}$; (b) and (c) are the prostate CT images warped by DMP-DVF and Demons-DVF; (d) Overlay of the warped prostate CT images in (a) and (b); (e) and (f) are the displacement errors (mm) of the DMP registration and the Demons registration overlaid on a sagittal view of the FEM constructed image$P_{1}^{CT}$.

**4. Discussion**

When adaptive radiation therapy is implemented in clinic, it becomes necessary to quantify the accuracy of its underlying deformable image registrations. In this study, we developed computational phantoms from lung cancer patient CT images to evaluate the performance of the DSP algorithm configured with a variety of B-spline grid setting options. The results revealed that the best performance of the DSP registrations was comparable to DMP. The DMP algorithm had average displacement errors ranging from 1.0 ~ 3.3 mm for the diaphragm compressions of 1 ~ 4 cm. This is comparable to the results of a multi-institution deformable registration accuracy study (MIDRAS)^16^. In the MIDRAS, the institutions that used the B-spline based registrations reported 1.6 to 3.0 mm errors when using a set of physician-identified landmarks as gold standards. In contrast to the landmark method, the computational phantom-based evaluation method uses simulated tissue deformation maps as benchmark models which allow DIRs to be evaluated in a voxel-by-voxel manner, and this method can overcome the limitations of the landmark-based evaluation such as observation uncertainty, lack of image features, or slice thickness induced round-off errors.

The accuracy of the DMP algorithm was found to be dependent on the magnitude of tissue deformation as well as individual patient images. As shown in Table 1, the registration errors increased for larger diaphragm deformations. This observation is similar to that reported by Liu *et al.* ^19^ where their in-house developed DIR algorithm was evaluated with a computational phantom (NCAT) and a physical liver phantom. However, the capability of their DIR algorithm could be underestimated because the NCAT phantom has uniform intensities assigned to each organ, and therefore, the DIRs performed on this phantom would not be as accurate as those performed on real patients. In contrast, the computational phantoms in this study, derived from actual patient CT images, do not have this limitation. It also further allows registration algorithms to be evaluated under patient-specific clinical scenarios.

With the patient-specific phantoms, the evaluation results showed that DMP registration outperformed Demons outside the lung, but inside the lung the DMP registrations were slightly worse. Both the DMP and Demons registrations demonstrated large errors in the lower lobe of the right lung and at the boundaries between the lung, diaphragm and chest wall, as marked in Figure 5c and 5d. At these boundaries, the simulated deformation fields were large, and such large deformation combined with the homogeneity of tissue could be a major source of registration errors ^39^.

For the prostate cases, DMP had smaller registration errors than Demons in the prostate. For the Demons algorithm, significant errors were observed in regions of low contrast tissue. With the simulated CBCT noise added, the accuracy of the Demons algorithm degraded significantly in all of the structures evaluated in this study, while the DMP algorithm remained robust. The average errors across the prostate, the rectum and the patient volume indicate that the simulated CBCT noise did not have a major impact on the DMP registration. This confirms the results from Murphy *et al.* where the simulated CBCT noise had no statistically significant impact on the B-Spline based image registrations ^37^.

It should be noted that, while different deformation scenarios can be simulated, limitations still exist for the computational phantom-based method. For example, on the 4D lung images it is common that motion artifacts differ among phase images. Further more, the differences of the organ filling, such as the rectum feces and gas, between the treatment planning CT and daily CBCT images creates one of the major challenges for deformable registrations. The impacts of such artifacts and differences have not yet been investigated. As a supplementary verification, the physical phantom was employed in this study to evaluate these DIR algorithms. However, the deformation of real patients is much more complex than the physical phantom deformation. Patient-specific verifications are still needed for clinical use of these DIR algorithms.

**5. CONCLUSIONS**

In this study, patient-specific FEM models have been developed and used as gold standard to evaluate the DIR algorithms implemented in the VelocityAI software package. It has been found that the accuracy of these algorithms is patient dependent, and related to various factors including the tissue deformation magnitudes and image intensity gradients across the regions of interest. This may suggest that the DIR algorithms need to be verified for individual deformation instances when implementing adaptive radiation therapy.

**ACKNOWLEDGMENTS:** This study is financially supported by NIH/NCI Grant No. R01CA140341.

Reference List

1 Frazier, A., Yan, D., Du, M., Wong, J., Vicini, F., Matter, R., Joyce, M., and Martinez, A., "Effects of treatment setup variation on beam's eye view dosimetry for radiation therapy using the multileaf collimator vs. the cerrobend block" Int.J.Radiat.Oncol.Biol Phys. 12-1-1995;33(5):1247-1256.

2 Frazier, R. C., Vicini, F. A., Sharpe, M. B., Yan, D., Fayad, J., Baglan, K. L., Kestin, L. L., Remouchamps, V. M., Martinez, A. A., and Wong, J. W., "Impact of breathing motion on whole breast radiotherapy: a dosimetric analysis using active breathing control" Int.J.Radiat.Oncol.Biol Phys. 3-15-2004;58(4):1041-1047.

3 Yan, D., Vicini, F., Wong, J., and Martinez, A., "Adaptive radiation therapy" Phys.Med.Biol 1997;42(1):123-132.

4 Kessler, M. L., "Image registration and data fusion in radiation therapy" Br.J.Radiol. 2006;79 Spec No 1S99-108.

5 Jenkinson, M. and Smith, S., "A global optimisation method for robust affine registration of brain images" Med.Image Anal. 2001;5(2):143-156.

6 Rohr, K., Stiehl, H. S., Sprengel, R., Buzug, T. M., Weese, J., and Kuhn, M. H., "Landmark-based elastic registration using approximating thin-plate splines" IEEE Trans.Med.Imaging 2001;20(6):526-534.

7 Rueckert, D., Aljabar, P., Heckemann, R. A., Hajnal, J. V., and Hammers, A., "Diffeomorphic registration using B-splines" Med.Image Comput.Comput.Assist.Interv. 2006;9(Pt 2):702-709.

8 Thirion, J. P., "Image matching as a diffusion process: an analogy with Maxwell's demons" Medical Image Analysis 1998;2(3):243-260.

9 Silva, J. S., Cancela, J., and Teixeira, L., "Fast volumetric registration method for tumor follow-up in pulmonary CT exams" J.Appl.Clin.Med.Phys. 2011;12(2):362-375.

10 Glocker, B., Paragios, N., Komodakis, N., Tziritas, G., and Navab, N., "Inter and intra-modal deformable registration: continuous deformations meet efficient optimal linear programming" Inf.Process Med.Imaging 2007;20: 408-420.

11 Rueckert, D., Sonoda, L. I., Hayes, C., Hill, D. L., Leach, M. O., and Hawkes, D. J., "Nonrigid registration using free-form deformations: application to breast MR images" IEEE Trans.Med.Imaging 1999;18(8):712-721.

12 Lu, W., Chen, M. L., Olivera, G. H., Ruchala, K. J., and Mackie, T. R., "Fast free-form deformable registration via calculus of variations" Phys.Med.Biol. 7-21-2004;49(14):3067-3087.

13 Fallone, B. G., Rivest, D. R., Riauka, T. A., and Murtha, A. D., "Assessment of a commercially available automatic deformable registration system" J.Appl.Clin.Med.Phys. 2010;11(3):3175-.

14 Bender, E. T. and Tome, W. A., "The utilization of consistency metrics for error analysis in deformable image registration" Phys.Med.Biol. 9-21-2009;54(18):5561-5577.

15 Hardcastle, N., Tome, W. A., Cannon, D. M., Brouwer, C. L., Wittendorp, P. W., Dogan, N., Guckenberger, M., Allaire, S., Mallya, Y., Kumar, P., Oechsner, M., Richter, A., Song, S., Myers, M., Polat, B., and Bzdusek, K., "A multi-institution evaluation of deformable image registration algorithms for automatic organ delineation in adaptive head and neck radiotherapy" Radiat.Oncol. 2012;790-.

16 Brock, K. K., "Results of a multi-institution deformable registration accuracy study (MIDRAS)" Int.J.Radiat.Oncol.Biol.Phys. 2-1-2010;76(2):583-596.

17 Castillo, R., Castillo, E., Guerra, R., Johnson, V. E., McPhail, T., Garg, A. K., and Guerrero, T., "A framework for evaluation of deformable image registration spatial accuracy using large landmark point sets" Phys.Med.Biol. 4-7-2009;54(7):1849-1870.

18 Kashani, R., Hub, M., Balter, J. M., Kessler, M. L., Dong, L., Zhang, L., Xing, L., Xie, Y., Hawkes, D., Schnabel, J. A., McClelland, J., Joshi, S., Chen, Q., and Lu, W., "Objective assessment of deformable image registration in radiotherapy: a multi-institution study" Med.Phys. 2008;35(12):5944-5953.

19 Liu, F., Hu, Y., Zhang, Q., Kincaid, R., Goodman, K. A., and Mageras, G. S., "Evaluation of deformable image registration and a motion model in CT images with limited features" Phys.Med.Biol 5-7-2012;57(9):2539-2554.

20 Vaman, C., Staub, D., Williamson, J., and Murphy, M. J., "A method to map errors in the deformable registration of 4DCT images" Med.Phys. 2010;37(11):5765-5776.

21 Schreibmann, E., Pantalone, P., Waller, A., and Fox, T., "A measure to evaluate deformable registration fields in clinical settings" J.Appl.Clin.Med.Phys. 2012;13(5):126-139.

22 Zhong, H., Peters, T., and Siebers, J. V., "FEM-based evaluation of deformable image registration for radiation therapy" Phys.Med.Biol. 8-21-2007;52(16):4721-4738.

23 Klein, A., Andersson, J., Ardekani, B. A., Ashburner, J., Avants, B., Chiang, M. C., Christensen, G. E., Collins, D. L., Gee, J., Hellier, P., Song, J. H., Jenkinson, M., Lepage, C., Rueckert, D., Thompson, P., Vercauteren, T., Woods, R. P., Mann, J. J., and Parsey, R. V., "Evaluation of 14 nonlinear deformation algorithms applied to human brain MRI registration" Neuroimage. 7-1-2009;46(3):786-802.

24 Heath, E. and Seuntjens, J., "A direct voxel tracking method for four-dimensional Monte Carlo dose calculations in deforming anatomy" Med.Phys. 2006;33(2):434-445.

25 Zhong, H. and Siebers, J. V., "Monte Carlo dose mapping on deforming anatomy" Phys.Med.Biol. 10-7-2009;54(19):5815-5830.

26 Salguero, F. J., Saleh-Sayah, N. K., Yan, C., and Siebers, J. V., "Estimation of three-dimensional intrinsic dosimetric uncertainties resulting from using deformable image registration for dose mapping" Med.Phys. 2011;38(1):343-353.

27 Lee, H. P., Lin, M. C., and Foskey, M., "Physically-based validation of deformable medical image registration" Med.Image Comput.Comput.Assist.Interv. 2008;11(Pt 2):830-838.

28 Zhong, H., Kim, J., and Chetty, I. J., "Analysis of deformable image registration accuracy using computational modeling" Med.Phys. 2010;37(3):970-979.

29 Wang, H., Dong, L., O'Daniel, J., Mohan, R., Garden, A. S., Ang, K. K., Kuban, D. A., Bonnen, M., Chang, J. Y., and Cheung, R., "Validation of an accelerated 'demons' algorithm for deformable image registration in radiation therapy" Physics in Medicine and Biology 6-21-2005;50(12):2887-2905.

30 Serban, M., Heath, E., Stroian, G., Collins, D. L., and Seuntjens, J., "A deformable phantom for 4D radiotherapy verification: design and image registration evaluation" Med.Phys. 2008;35(3):1094-1102.

31 Chang, J., Suh, T. S., and Lee, D. S., "Development of a deformable lung phantom for the evaluation of deformable registration" J.Appl.Clin.Med.Phys. 2010;11(1):281-286.

32 Kerdok, A. E., Cotin, S. M., Ottensmeyer, M. P., Galea, A. M., Howe, R. D., and Dawson, S. L., "Truth cube: establishing physical standards for soft tissue simulation" Med.Image Anal. 2003;7(3):283-291.

33 Nie, K., Chuang, C., Kirby, N., Braunstein, S., and Pouliot, J., "Site-specific deformable imaging registration algorithm selection using patient-based simulated deformations" Med.Phys. 2013;40(4):041911-.

34 Varadhan, R., Karangelis, G., Krishnan, K., and Hui, S., "A framework for deformable image registration validation in radiotherapy clinical applications" J.Appl.Clin.Med.Phys. 2013;14(1):192-213.

35 Zhong, H., Jin, J. Y., Ajlouni, M., Movsas, B., and Chetty, I. J., "Measurement of regional compliance using 4DCT images for assessment of radiation treatment" Med.Phys. 2011;38(3):1567-1578.

36 Nesetril, J., Milkova, E., Nesetrilova, H., and Otakar B., "On minimum spanning tree problem" Discrete Mathematics 2001;2333-36.

37 Murphy, M. J., Wei, Z., Fatyga, M., Williamson, J., Anscher, M., Wallace, T., and Weiss, E., "How does CT image noise affect 3D deformable image registration for image-guided radiotherapy planning?" Med.Phys. 2008;35(3):1145-1153.

38 Jaffray, D. A. and Siewerdsen, J. H., "Cone-beam computed tomography with a flat-panel imager: initial performance characterization" Med.Phys. 2000;27(6):1311-1323.

39 Zhong, H., Kim, J., Li, H., Nurushev, T., Movsas, B., and Chetty, I. J., "A finite element method to correct deformable image registration errors in low-contrast regions" Phys.Med.Biol 6-7-2012;57(11):3499-3515.

Figure 1. The original CT image (red) and its deformed image (green) of Lung1_2cm_. (a), (b) and (c) are the coronal, sagittal and transverse cuts of their overlaid images, respectively.

Figure 2. Average displacement errors (± standard deviation) for different B-spline grid settings. R_x_ denotes a DSP registration with the grid resolution x, and R(x🡪y) represents the composition of the DSP registrations with the resolution chain x🡪y.

Figure 3. Superior-inferior (SI) displacement profiles derived from DMP registrations for Lung1. (a) The profiles contain displacements from the gold standard DVFs and DMP DVFs at diaphragm deformation of 1, 2 and 3cm; (b) the SI line illustrated in Lung1’s primary CT image is corresponding to the displacement profiles in (a). The tumor region is marked with two red lines on both the image and profile figures.

Figure 4. Computational lung phantom Lung4 with large areas of homogeneous tissue: (a) the original image set, (b) the FEM simulated image set, (c) image warped by Demons DVF, and (d) image warped by DMP DVF.

Figure 5. Color overlay of displacement errors with CT image sets. The figure contains an axial cut of image set Lung2_2.7cm_ overlaid with the errors of (a) DMP registration and (b) Demons registration. Also shown is a coronal slice of Lung1_3cm_ overlaid with (c) DMP and (d) Demons registration errors. Error values seen in the color bar (e) are given in mm.

Figure 6. Physical phantom image sets. (a) A picture of the physical phantom; the overlay of EE and warped EI images registered with (b) 250 window setting and (c) 500 window setting. The green image is the EE image and the red one is the warped EI image. The large circular object represents the tumor.

Figure 7. Computational prostate phantom and simulated CBCT image sets. The axial cuts of (a) the original image set, (b) the deformed image set and (c) the deformed image with simulated CBCT noise added. The subfigures (d-f) are the coronal views of (a-c), respectively.

Figure 8. (a) the FEM constructed image$P_{1}^{CT}$; (b) and (c) are the prostate CT images warped by DMP-DVF and Demons-DVF; (d) Overlay of the warped prostate CT images in (a) and (b); (e) and (f) are the displacement errors (mm) of the DMP registration and the Demons registration overlaid on a sagittal view of the FEM constructed image$P_{1}^{CT}$.

Table 1. Average displacement errors (mm) and standard deviation for DMP and Demons registrations evaluated with computational phantoms of different deformation magnitudes.

| Image set (motion magnitude) | Patient volume | | Lung | |
| --- | --- | --- | --- | --- |
|  | DMP | Demons | DMP | Demons |
| Lung1 (10 mm) | 1.0 ± 0.7 | 0.8 ± 0.5 | 0.9 ± 0.5 | 0.7 ± 0.4 |
| Lung1 (20 mm) | 1.1 ± 0.8 | 0.9 ± 0.7 | 0.9 ± 0.6 | 0.7 ± 0.4 |
| Lung1 (30 mm) | 1.3 ± 1.1 | 1.1 ± 1.0 | 1.0 ± 0.7 | 0.8 ± 0.6 |
| Lung1 (40 mm) | 1.6 ± 1.5 | 1.4 ± 1.6 | 1.2 ± 1.1 | 1.1 ± 1.2 |
| Lung2 (18 mm) | 2.1 ± 0.8 | 5.2 ± 5.7 | 2.1 ± 0.8 | 2.1 ± 0.9 |
| Lung2 (27 mm) | 2.2 ± 1.2 | 5.2 ± 5.4 | 2.4 ± 1.1 | 2.3 ± 1.2 |
| Lung2 (42 mm) | 3.1± 1.7 | 5.9± 5.6 | 3.3± 2.1 | 3.0± 1.4 |

Table 2. Average displacement errors (mm) and standard deviations for DMP and Demons registrations evaluated with different patient images.

| Image set (motion magnitude) | Patient volume | | Lung | |
| --- | --- | --- | --- | --- |
|  | DMP | Demons | DMP | Demons |
| Lung1 (30 mm) | 1.3 ± 1.1 | 1.1 ± 1.0 | 1.0 ± 0.7 | 0.8 ± 0.6 |
| Lung2 (27 mm) | 2.2 ± 1.2 | 5.2 ± 5.4 | 2.4 ± 1.1 | 2.3 ± 1.2 |
| Lung3 (30 mm) | 2.6 ± 1.7 | 2.1 ± 1.3 | 2.4 ± 1.3 | 1.9 ± 0.7 |
| Lung4 (30 mm) | 2.4 ± 2.3 | 30.0 ± 18.1 | 1.7 ± 1.0 | 9.3 ± 6.3 |

Table 3. Average displacement errors and standard deviations of DMP registrations

| Mean Error (mm) | DMP registration | | | | |
| --- | --- | --- | --- | --- | --- |
| Intensity Window | 250 | 500 | 1000 | 1500 | Unlimited |
| Image domain | 1.3 ± 0.6 | 1.3 ± 0.5 | 1.1 ± 0.5 | 1.3 ± 0.7 | 1.9 ± 0.8 |
| Lung region | 0.8 ± 0.4 | 0.8 ± 0.4 | 1.2 ± 0.7 | 1.1 ± 0.5 | 1.8 ± 0.6 |

Table 4. Average displacement errors (mm) and standard deviation for DMP and Demons registrations for pelvic images .

|  | Whole Patient | | Prostate | | Rectum | | Bladder | |
| --- | --- | --- | --- | --- | --- | --- | --- | --- |
| Image Set | DMP | Demons | DMP | Demons | DMP | Demons | DMP | Demons |
| $P_{1}^{CT}$ | 2.1 ± 0.7 | 10.3±11.5 | 1.9 ± 0.5 | 3.2 ± 1.2 | 1.9 ± 0.5 | 2.2 ± 0.9 | 2.8 ± 1.5 | 3.7 ± 1.8 |
| $P_{2}^{CT}$ | 1.9 ± 1.0 | 7.8 ± 8.3 | 1.2 ± 0.6 | 2.4 ± 1.3 | 2.0 ± 0.8 | 2.4 ± 1.0 | 4.6 ± 3.1 | 5.0 ± 3.4 |
| $P_{1}^{Noise}$ | 2.1 ± 0.8 | 18.5±18.1 | 1.7 ± 0.5 | 12.3±10.5 | 1.8 ± 0.6 | 12.8±11.2 | 3.1 ± 1.7 | 16.3±13.9 |
| $P_{2}^{Noise}$ | 1.8 ± 0.9 | 19.4±20.7 | 1.0 ± 0.6 | 13.2±11.7 | 2.4 ± 1.0 | 13.3±12.0 | 5.0 ± 3.4 | 18.3±14.5 |
